# The Allied Health Expansion Program: Rethinking how to prepare a workforce to enable improved public health outcomes

**DOI:** 10.3389/fpubh.2023.1119726

**Published:** 2023-02-17

**Authors:** Lisa M. Dalton, Andrew P. Hills, Sisitha Jayasinghe, Kendra Strong, Paula Hyland, Nuala M. Byrne

**Affiliations:** ^1^College of Health and Medicine, School of Health Sciences, University of Tasmania, Launceston, TAS, Australia; ^2^Department of Health, Tasmanian Government, Hobart, TAS, Australia; ^3^Tasmanian Health Services, Department of Health, Hobart, TAS, Australia

**Keywords:** allied health, education, public health, workforce, Tasmania

## Abstract

Improvements in global public health require universal health care supported by a health workforce with competencies appropriate for local population needs–the right capabilities, in the right place, and at the right time. Health inequities persist in Tasmania, and Australia more broadly, most notably for those people living in rural and remote areas. The article describes the curriculum design thinking approach being used to codesign and develop a connected system of education and training to target intergenerational change in the allied health (AH) workforce capacity in Tasmania, and beyond. A curriculum design thinking process is engaging AH participant groups (faculty, AH professionals, and leaders across health, education, aged and disability sectors) in a series of focus groups and workshops. The design process deals with four questions: *What is? What if? What wows?* and *What works?* It also involves *Discover, Define, Develop* and *Deliver* phases that continue to inform the development of the new suite of AH education programs. The British Design Council's Double Diamond model is used to organize and interpret stakeholder input. During the initial design thinking *discover* phase, stakeholders identified four overarching problems: rurality, workforce challenges, graduate skill set shortfalls, and clinical placements and supervision. These problems are described in terms of relevance to the contextual learning environment in which AH education innovation is occurring. The *develop* phase of design thinking continues to involve working collaboratively with stakeholders to codesign potential solutions. Solutions to date include AH advocacy, a transformative visionary curriculum, and an interprofessional community-based education model. In Tasmania, innovative educational innovations are catalyzing attention and investment in the effective preparation of AH professionals for practice to deliver improved public health outcomes. A suite of AH education that is deeply networked and engaged with Tasmanian communities is being developed to drive transformational public health outcomes. These programs are playing an important role in strengthening the supply of allied health professionals with the right capabilities for metropolitan, regional, rural, and remote Tasmania. They are situated in a broader AH education and training strategy that supports the ongoing development of the AH workforce to better meet the therapy needs of people in Tasmanian communities.

## Background and rationale

The interconnected nature of the modern world has increased the interest and investment in global public health (1). Whilst the World Health Organization considers the health workforce to be critical to achieving public health, Australia's health system is facing significant challenges (2) and universal access to all health professionals is commonly not possible for all communities (1, 3). This is particularly apparent in Tasmania, a small island state of Australia. The problems associated with public health in Tasmania are threefold: our health and wellbeing outcomes linger behind the rest of Australia; the current organization and delivery of allied health services are inadequate for addressing the state's public health challenges; and allied health education and training is insufficient to create an appropriate local health workforce.

Tasmania has a decentralized population of 541,000 is growing and aging, with over 25% of people having a disability, 17.7% higher than the national average (4). The health and wellbeing of large sections of the Tasmanian community are subpar, and in some cases, in dire straits. Tasmanians consistently report low levels of self-assessed health, have a lower life expectancy, higher infant mortality rates and are more susceptible to developing chronic disease during their lifetime compared to mainland Australians (5). Considerable social disadvantage, including disengaged youth, unemployment and low income, and contact with the criminal justice system, concentrates in communities outside Hobart and Launceston (6). Reasons for the current predicament vary across different patterns of inequity that exist in income, education and aspiration but can be predominately linked to poor access to health services and strategies for prevention of chronic disease (7).

Tasmania, like much of rural and regional Australia, faces chronic challenges in recruiting and retaining health professionals (3). For AH in particular, current labor market data indicates significant shortages and difficulties recruiting staff with the appropriate skillsets and experience (7). In 2018, ~4,000 nursing and AH positions were advertised in Tasmania for about 5,000 vacancies (8). Nationally, there is a recognized AH workforce geographic maldistribution. National workforce data shows the number of AH professionals available per capita remains lower in regional and remote areas than metropolitan cities and most are working privately in affluent areas than in lower socioeconomic areas (3). This means that despite a higher prevalence of both aging populations and chronic conditions per capita in rural areas, there are fewer allied health professionals in these needy areas of Tasmania than in the healthier urban areas (7). Against this backdrop, health and wellbeing in Tasmania is expected to worsen as the COVID-19 pandemic continues to impact our communities. Not only did the pandemic expose the weaknesses in rural healthcare, its influence on mental health and wellbeing was recognized early (9). Communities remain concerned about contracting the virus, loss of social interaction, restriction to movement, transition to remote work or study and financial impacts and are now dealing with exacerbation of pre-existing mental health conditions (9).

Compounding public health problems in Tasmania, is that while the University of Tasmania is the sole university in the state graduating health professionals it was not equitably servicing all state regions or health disciplines. In 2019 UTAS was not offering many AH degrees needed to gain the qualifications necessary to be eligible for certification or registration as a health professional in Australia. Collectively, health service access limitations, a maldistributed AH workforce, skillset gaps and limitations in AH education and training options place Tasmania at a distinct disadvantage in terms of chronic disease treatment and management relative to the rest of Australia. Our hypothesis is that by collaborating with government, health professionals, industry and local Tasmanian communities, we believe it is possible to create opportunities to better support the health labor force needs in Tasmania, solving problems associated with the distribution, quality, and performance of Tasmania's AH workforce. We expect to see increased AH education, training and research opportunities across different regions of Tasmania that will lead to improved public health outcomes and health system transformation.

The Australian Academy of Health and Medical Sciences aims to better integrate health and medical research and innovation within the health system for evidence based and research informed system transformation (10). However, to fully realize the vision to transform public health outcomes for the Australian community, a shift in the purpose of higher education is also required (11). Higher education is critical to advancing universal health care and the Sustainable Development Goals through preparing health professionals for 21st-century practice. Our higher education systems supply graduate health professionals, and shape the distribution, quality, and performance of the available health workforce (12). Some pedagogies, such as experiential learning, inquiry-led learning and problem based learning have proven effective in equipping public health students with applied skills and opportunities for application to respond to local population health and wellbeing needs (13). If Tasmania is to succeed in improving public health and transforming its health system, the state requires all health graduates to have training in public health supported by educational programs that respond to the role that the pandemic has played in accelerating the need for improvements in blended and online delivery (14). Educational reform and innovation is therefore required to catalyze investment in the effective preparation of all health professionals for practice to deliver improved public health outcomes (3). The University of Tasmania (UTAS) is leading strategic initiatives to concurrently improve health care services, build capacity in communities and work sustainably while delivering on educational innovation.

For many years, the Australian government has funded a range of health professional education initiatives as part of its strategy to build a sustainable, high-quality health workforce that is distributed across the country according to community need (15, 16). Some of these initiatives include rural clinical schools, increased selection and support for rural background students, and financial support for students to train in rural and remote communities *via* a network of training facilities (16). For Tasmania, these supports have enabled the UTAS to increase the number of medical and nursing graduates in regional communities to benefit some rural communities (17). AH has not benefited to the same extent as the medical and nursing professions. Compared to Australia as a whole, Tasmania has more nurses per 100,000 population (18) and comparable to the national average the density of medical practitioner FTE to population is 430 per 100,000 population (7).

The Australian government recently examined priorities for improving the access, distribution, and quality of rural and remote AH services to develop a national policy and investment response for the rural AH workforce (3). The national aspirations augment the goals and aspirations of the Healthy Tasmania Five-Year Strategic Plan 2022–2026 (7). The state health plan articulates the Tasmanian government's vision for strengthening preventive health in Tasmania and brings together communities, services, and all levels of government to work in partnership for improved health and wellbeing (7). For Tasmania, this means appropriately prioritizing effort toward health promotion and development of a health workforce spanning the state that can support the primary, secondary and tertiary prevention of chronic disease. Accordingly, all schools (nursing, midwifery, medicine, paramedicine, psychology, public health, medical sciences, exercise science and physiology, nutrition sciences and laboratory medicine) within the College of Health and Medicine are now developing and embedding distinctive, sustainable curricula across their programs to create agile leaders in health and accelerate discovery and translational research capacity. The article describes a strategic and evidence-based approach the School of Health Sciences (SHS) in the College of Health & Medicine commenced in 2019 to organize AH workforce education and development in a way that puts public health at the center with responsibility shared amongst major stakeholders including the university, health services and the health professions.

## The Allied Health Expansion Program (AHEP)

In 2019, the AHEP was launched by UTAS as a major strategic initiative to increase AH education and workforce development opportunities in Tasmania and will continue until 2029. The goal of the AHEP is to strengthen the AH education system to develop a well-performing, stable, and equitably distributed workforce with an appropriate mix of skills to concurrently improve health care services, build capacity in communities and work sustainably while delivering on educational innovation.

Objectives are to:

- increase Tasmanian's interest in AH careers and access to AH courses that cannot be sustainably offered by UTAS
- provide the Tasmanian community with access to innovation in learning about preventative health and health promotion to develop self-health capability and generate interest in AH careers
- codesign, develop and offer a suite of new AH degrees that are viable, sustainable, prepare graduates for practice that transforms the health system and public health outcomes, and
- provide the currently available Tasmanian AH workforce with access to convenient, industry-relevant professional development that can be applied in everyday work practices, and build skill sets to enhance current and future career ambitions.

Whilst the university program initiatives are led by a team of UTAS AH academics, the overall AH workforce education and development strategy is a collaboration with government, health professionals, industry, and local communities.

## Pedagogical framework: Curriculum design thinking

Globally, most governments are aspiring to develop healthy populations (1). For example, the United States articulates a Healthy People 2030 (19) that has an overarching vision for “a society in which all people can achieve their full potential for health and wellbeing across the lifespan”. Likewise, Australia has a long term national health plan in place to build the world's best health system to improve the health and wellbeing of Australian citizens (15). However, this is particularly challenged by approximately 7 million people, or 28% of the Australian population, living in rural and remote areas (4). Educating Australian health professionals to address universal health care and the social determinants of health in and with communities requires curricula that aligns with community needs (3). In the early 2000's, the hallmarks of exemplary Australian health professional education programs (20) were identified as:

- commitment to multidisciplinary and community-based education,
- community-based placements,
- formal linkages with government entities, and
- a structured approach to community participation.

The pedagogical framework for the AHEP curricula was based on best practice approaches in the Scholarship of Learning and Teaching and was largely drawn from the vast experience of Schools of Public Health engaged in education of graduates who are prepared to improve health through a population health focus (21). Within public health education there is general consensus that to advance toward addressing the complex, systemic public health problems future health professionals must be equipped with leadership and interprofessional skills that support collaboration and a culture of health (21). Public health curricula is typically characterized by integration, problem based learning and embedded practice experiences, which are essential components of all Australian AH university courses to meet accreditation requirements (22). AHEP was also designed at a time when the COVID-19 pandemic had forced health schools to close their campuses and move online delivery, therefore “online and digital innovation, discipline economic viability and clearly defined operating structures” (14) were important priorities in the pedagogical framework.

Australia has decades of experience in establishing rural, community-engaged health professional schools and higher education initiatives that embrace active community participation, curricula that meets community needs and advance national and international health equity agendas (20). The need for community engagement that is locally sensitive and ensures community leaders recognize the value of engaging with universities through honest and trustful dialogue is consistently evidenced (20). These learnings directly align with the concept of *innovative learning environments* (ILEs), which has been applied by the Organization for Economic Co-operation and Development (OECD) in their ILE framework (23). ILEs value systems that are based on the social nature of learning and assume collaborative arrangements with a range of partners (23).

For AHEP, we envisaged a curriculum that could be *experienced* by learners and not just a program of study that is *enacted* by educators on campus and in practice settings. Following Dewey (24), we did not consider knowledge as a thing-in-itself but instead a transactional construction and a function of inquiry. Thus, as Australian AH accreditation requirements stipulate, the occupation-specific subject matter is centralized as the intended formal curriculum (25), however the programs are also designed to engender informal and hidden curriculum elements (25) that continually reinforce a rich theory of inquiry (24), interprofessional collaboration (26), and public health to intentionally facilitate the transactional relationship that exists between AH students as inquirers and the social world of AH practice situated in different Tasmanian communities. The pedagogical framework therefore incorporates three conceptions of AH curriculum (27):

- **Intended curriculum**—the planned program syllabus underpinned by clear educational philosophies, and program aims set out in course level learning outcomes;
- **Enacted curriculum**—the way AH educators and professionals who supervise students in practice settings enact the curriculum based on their interpretation of what the curriculum is, and
- **Experienced curriculum**—what AH students experience as they traverse their study program.

Curriculum design thinking provides a participatory approach to the design, build and delivery process the AHEP requires. Both an ideology and a process, design thinking is simply a way of working with stakeholders using a human-centered problem-solving process to collaboratively solve wicked problems (28). Curriculum design thinking involves the application of this approach to engage community, students, health professionals and health industry employers, as end users, in the coproduction of learner-centered education (29). Considering changes needed in the Tasmanian distribution, quality, and performance of the AH workforce, curriculum design thinking offers a way to lead educational innovation and catalyze attention and investment in effective AH workforce education and development to deliver improved public health outcomes and health system transformation.

## Allied health learning environment and curriculum design thinking approach

The British Design Council's Updated Double Diamond model (30), comprising *Discover, Define, Develop* and *Deliver* guides the curriculum design thinking process. The four phases are not sequential but are instead considered as different modes that contribute to the entire design project. Unlike the more traditional deductive approach to curriculum design, a design-driven approach relies on abduction method (31, 32). The only known variable in our approach is the Value (32): improving Tasmanians access to AH services by offering local AH workforce solutions *via* new AH education and training options. The What (32) (Curriculum) and How (32) (Course Type, Delivery Model, Staffing, Delivery Locations, Clinical Placements) are components of the education system being approached as unknowns in need of investigation (30). Accordingly, the curriculum design thinking process deals with four questions, which corresponded to four stages of the AHEP design, build and delivery process: *What is? What if? What wows?* and *What works?* (33).

The “*What is?”* (33) stage involves empathizing with the difficulties and challenges that Tasmanian AH professionals, health facilities, and community members experience in accessing and delivering health services. In this paper, we focus on the findings of the extensive participatory stakeholder engagement exploring what it means to deliver AH services in Tasmania and how those conditions influence the AHEP innovative learning environment. Valuing “end user” perspectives (34) allows us to harness valuable holistic insight about workforce needs, resourcing, and pragmatic issues that challenge health service delivery in Tasmania and consider their ramifications for innovating our new AH education programs.

To envision a new future, the “*What if?”* (33) exploration involves thinking and reasoning with stakeholders to form hypotheses drawn from incomplete sets of information. All possibilities are considered as opportunities to codesign a clear strategy to concurrently improve health care services, build capacity in communities and work sustainably while delivering new AH programs. This period of retrograde analysis includes a problem-solving process that oscillates between abductive logic (34) to consider what might be in order to make inferences about each of the identified problems and inductive logic (34) to draw generalized conclusions about each of those problems.

At the “*What wows?”* (33) stage, discussions and debates work to “stimulate the imaginations” (34) of our stakeholders to codesign new possibilities using an attitude of solution-based thinking. The “*What works?”* (33) phase involves critical analysis, assessment, and modeling to ascertain what is required to act and test the possible solutions. At this point we balance what is desirable from our stakeholder's point of view with what is logical, feasible and economically viable for the university to deliver. As AHEP continues to progress, the transition from “*What is?”* to “*What works?”* (33) is iterative, ongoing and involves various cycles of rethinking and refining to guide the process of untangling the unknowns to become knowns. Several overarching problems have already been discovered and defined and we are now developing, testing, and refining viable ideas and working through whether weak or unviable ideas should be abandoned or rejigged.

## Educating allied health professionals to address public health: Challenges, pressures and solutions

**Figure 1:**
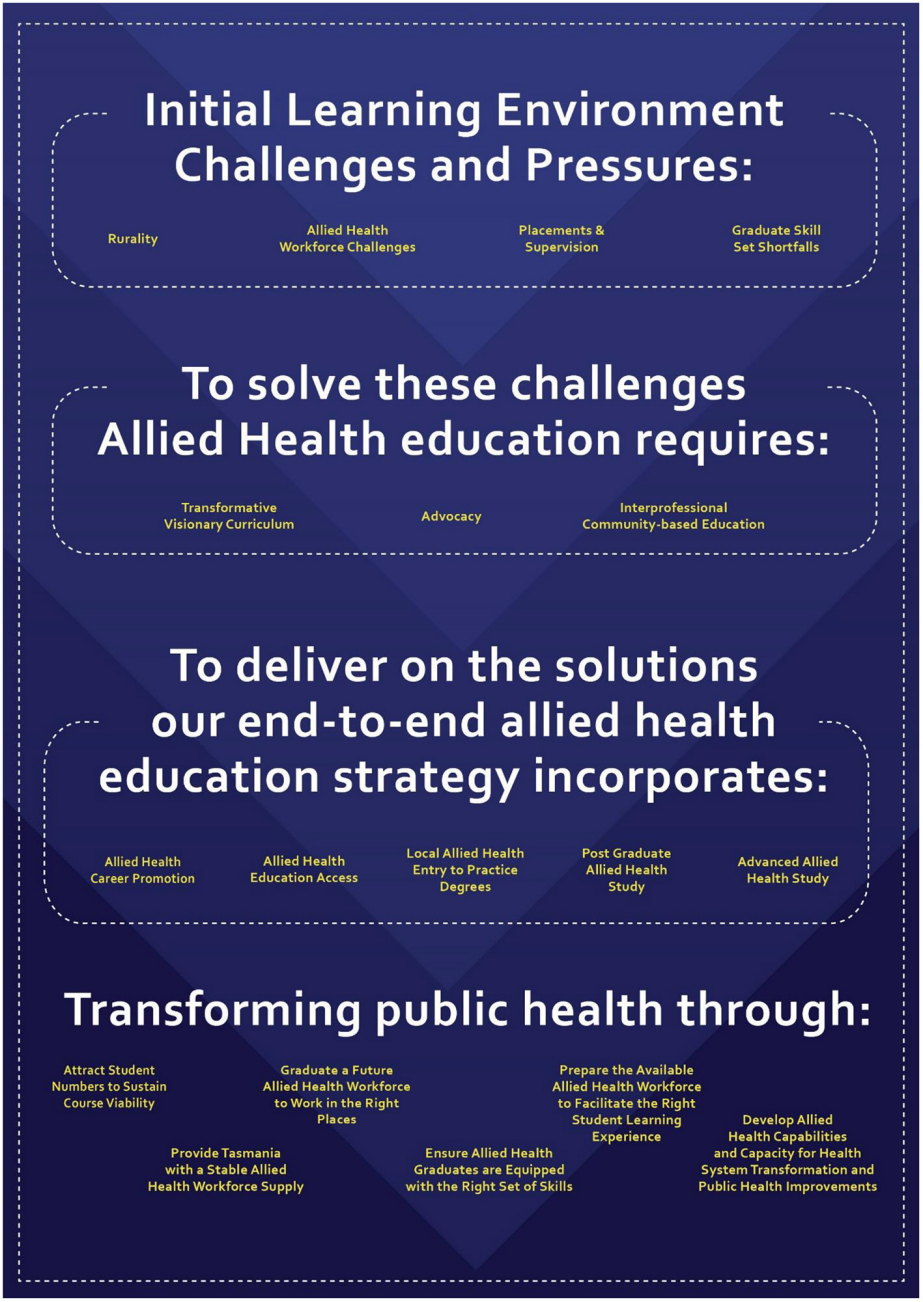
AHEP challenges, pressures, and solutions to transform public health.

In line with the Updated Double Diamond model (30), the *Discovery* and *Define* phases of the curriculum design thinking process led to the identification of problem themes that allowed stakeholders work together to deeply and wholistically understand the challenges and pressures affecting the innovative learning environment for AH education in Tasmania. Component parts were isolated to focus the codesigning of potential solutions (Figure 1).

There were four overarching problems affecting the innovative learning environment. First, the rural nature of Tasmania means many people leave to study AH in other states, there is an underrepresentation of rural origin students in higher education, and for some rural communities intermittent and service gaps are challenging their ability to access timely AH services. Second, the serious AH workforce challenges mean there are long wait lists and health facilities are short staffed, experiencing recruitment and retention challenges and are reporting gaps and variations in skills mix. The university faces similar challenges in building an academic AH workforce. The problems associated with Tasmania's rural topography and AH workforce issues combine to give rise to a third set of problems related to placements and supervision.

Many universities use Tasmania to place students for work-based experiential learning. While some use long-arm supervision models most rely on AH professionals in public and private practice. These long-established partnerships are valued in Tasmania. AH professionals want to maintain relationships with other universities to support Tasmanian students already studying interstate and to access diverse research and curriculum. Nevertheless, increasing the number of local AH students is increasing demand for statewide placements across a range of practice areas. Fourth, through their engagements with supporting placements and supervision, the Tasmanian AH professionals note some students do not seem well prepared for the landscape of health service delivery changing from siloed, fragmented and disease-centered toward integrated, people-centered care and requiring more effective interprofessional collaboration.

The *Develop* and *Deliver* (30) phases of the curriculum design thinking process are iterative and ongoing to codesign potential solutions with stakeholders and implement initiatives in ways that ensure program viability and sustainability. Advocacy for AH is ongoing and includes direct lobbying through communications and meetings with government representatives and agencies, and by ensuring AH academics, leaders and practitioners are represented on university advisory groups. Collaboration between UTAS and the Departments of Health (DoH) and Education occurs when making submissions to government and other funding bodies. Both the DoH and the UTAS are proactively investing in and attracting resources to strategic projects that address key AH issues identified during the discovery and define phases of the AHEP.

UTAS is now developing new pathway programs to facilitate prospective applicants in successfully meeting AH course entry requirements. Course offerings now include a new Master of Physiotherapy, a new Master of Speech Pathology and the university is working toward developing degrees in Occupational Therapy and Clinical Exercise Physiology. Each degree is using an interprofessional-community-based education model to deliver a transformative visionary curriculum that is carefully designed to better equip AH graduates to improve public health, safely respond to complexity and uncertainty, and contribute to health system transformation. There is substantial evidence that rural community-based medical education programs can facilitate effective relationships between students, practitioners, clients (35), involve community (36) and improve students understanding of the social determinants of health (26). In turn, these relationships are known to influence students' competency acquisition and professional identity, increase graduates' interest in rural careers, and improve rural health service delivery (37).

Designing programs to prepare for the unpredictability of practice is challenging when curriculum must also respond to various education drivers—technology, policy, competency standards, accreditation standards, evolving evidence. Transformative education is the educational philosophy underpinning the AHEP degrees. It offers new possibilities for curriculum to generate AH practice transformation toward renewed values of health equity and social justice to address social inequities in health (38) and allows for an androgogy of uncertainty that acknowledges the uncertain and complex nature of professional practice (39).

Primary health care is the program philosophy to ensure the underlying concepts of the social model health and disability, and the principles of universal health care and social determinants of health, as they relate to clients, health conditions, and service delivery, are centralized in the curriculum. The program philosophy is explicitly and consistently enacted as the program ontology to continually shape AH values, norms and practice approaches that are of relevance to the social determinants of health. The curriculum ontology is one of the most important design features used to organize knowledge in the UTAS AH programs. It is a powerful tool that allows curriculum knowledge to be structured to underscore the key points of knowledge (40), namely public health, primary health care and interprofessional collaboration –the hallmarks of preparing graduates for 21st-century healthcare practice.

At the time of program design, Australian universities had been forced to move all programs to online delivery models due to domestic travel and social movement restrictions (14). To accelerate and facilitate digital delivery we required cost effective mechanisms to innovate, rapidly develop and implement new online learning opportunities. The AH degrees were therefore developed as a suite and the build process was progressed by a specialist team with clear role delineations and responsibilities. Discipline specific academics, as the key content developers, co-created raw materials and designed learning activities with AH students and AH professionals. Educational designers transformed raw content and learning activities to align the learning experience to course and unit learning outcomes and objectives, assessments, and evaluation criteria. The educational technologists acted as engineers who determined which digital tools were needed and then built a functional and engaging learning program for delivery. The specialist team and coordinated approach to multi-program development enabled SHS to capitalize on shareable content, streamline the digital infrastructure build and use a lean workflow plan for program development.

A flipped curriculum (41) is being used as the educational approach to engage students in active, dynamic and proactive learning activities where they engage in various forms of interaction, undertake practical learning tasks and enact autonomy in their learning experience. The approach is based on the flipped classroom derived from Dewey's (24) theory of inquiry and it means that educational experiences that traditionally took place inside the classroom now take place in other learning environments (42), including online learning environments, on-campus classrooms, health care facilities and community environments. Our early approach to flipping classrooms involved creating learning activities that facilitate *engagement, exploration*, and *explanation* (43)in the digital learning environment and then linking those learnings to activities so that students can *consolidate* (43) knowledge and skills in classrooms or professional practice learning environments. However, to achieve coherence across the entire curriculum we soon identified this approach required a broader application beyond selected classrooms. Dewey's (24) educational philosophy demands that classrooms are not merely flipped, but that entire curricula is flipped (41). Instead of isolated classroom situations requiring students to demonstrate a quantitative increase of facts or skills, we now ensure the full program of study can offer students an ongoing process of personal and cultural growth and maturation (i.e. a process of professional transformation in which the ontological values of public health, primary health care and interprofessional collaboration are continually adopted, extended and enacted in practice) (24, 41).

For AHEP, the value of the flipped curriculum architecture of education is twofold. First, it allows students to directly experience the kinds of ambiguous, value-laden, and relationally complex problems that are constitutive of rural community-based AH practice and the very practices of inquiry that constitute each of the AH disciplines, and the multidisciplinary nature of health care. Second, it provides a strong impetus for the program design, build and delivery teams to find new ways to ensure students remain engaged and activated throughout the whole program by integrating pedagogical technology at every stage of the model: online, on campus, and during experiential learning placements.

In working toward solving some of the challenges and pressures associated with clinical placements and supervision, UTAS is developing of a network of student assisted multidisciplinary AH clinics across regional and rural Tasmania. These models offer an innovative approach to expand healthcare access and equity and build clinical placement capacity for health professional students (44).

The DoH acknowledges that the AH workforce is integral to Tasmania's health, education, disability and aged care services and how the lack of a local training program for most AH professions exacerbates local recruitment and retention issues. The Department is therefore proactive in supporting initiatives that may lead to increased supply of AH professionals and is committed to collaborating with the university to lead cultural changes to ensure the public health system can provide a suitable educational environment that meets the needs of all learners. A successful funding bid is enabling new Clinical Lead—Education and Support roles to be established across the three priority AH areas in the North, North-West and South of Tasmania. Such leadership in education, underpinned by leadership engagement, measures and feedback and clinical targets is a critical element to develop a workplace learning culture (45). The funding is also supporting rapid upskilling of AH professionals in supervision-related skills and capacity across the DoH and Department of Education, Children and Young People (DECYP). Educational delivery that is underpinned by quality supervision, valuing learning, and effective resourcing is another important component of our approach to strengthening the workplace learning culture (45). There are already tangible signs that the collaboration between the DoH and the UTAS is effective and as the partnership continues it is expected that integrated learning will become embedded in AH working practices and the shared vision for AH education in Tasmania will be fully realized.

## Practical implications and constraints

Public health is everybody's business. It operates at every level and matters at individual, community society, and global levels (1). There is tangible agreement that UTAS, DoH, Tasmanian health system, health services and the health workforces need to work together to support the health and wellbeing needs of all Tasmanians, and address the complexities associated with persistent chronic diseases and injuries. Our curriculum design thinking approach evidences that while some alterations to the structure and delivery of AH education is warranted, the need to achieve a long-term, whole system change for AH education and workforce development, supply and distribution is extremely challenging. Returning to the *What* and *How* (32) in curriculum design, the design thinking process is proving to be critical for UTAS to design and deliver solutions to achieve the *Value* (32) variable: improving Tasmanians access to AH services by offering local AH workforce solutions *via* new AH education and training options. It is also enabling a range of Tasmanian AH stakeholders to have input into the process of codesigning the *How* (32) variable: advocating and influencing state strategic priorities to ensure resource allocation can be attracted and invested in AH initiatives; establishing an end-to-end interprofessional community-based education model that attracts, prepares and extends AH professionals across a career continuum, creation of a suite of new AH programs, and student assisted multidisciplinary clinics. Amidst the complexity, however, it is the *What* (32) variable that has possibly been the most challenging.

Our findings evidence the way our AH stakeholders, like others in health (46), are making calls for education programs to move beyond curriculum that prepare graduates for practice in large city centers with a primary focus on acute care orientated in the prevailing biomedical model that tends to dominate healthcare. The United Nations Educational, Scientific and Cultural Organization (UNESCO) Education 2030 Agenda and Framework for Action, in particular Target 4.7 of Sustainable Development Goal 4: Quality Education (47), calls on higher education to be concurrently responsible for shaping more peaceful, tolerant, and inclusive societies. Health professional education that focuses on the human condition and targets treatment on body parts overlooks the social and psychological sources of most healthcare problems (46). Globally, there are calls for a more meaningful emphasis on public health (1), with the key point being as that as important as good health care is it is now time to place more emphasis on people's health and wellbeing (2). There is a growing consensus that all health professional graduates must be better prepared for more consideration of the social determinants of health so the health of the world's population can be sustainably improved (2).

Our stakeholders identified the need to bolster programmatic and AH professional capacities with a broader set of skills and knowledge that support the multi-sector vision and leadership needed to be agile and responsive as health care continues to change and evolve. WHO acknowledges that it is time to professionalize all the health workforce as part of the public health workforce (2). In rethinking the *What* (32) in AH curriculum we committed to the dual focus of producing profession-ready graduates and equipping them with future facing capabilities required to adapt and respond to rapidly occurring changes in health, and the health system. This means we are carefully tackling the question of *what else* needs to be taught to enable improved public health outcomes, and *how* it needs to be taught. UTAS is opening new ways of learning and teaching that develop in students a sense of belonging to a much wider community than their chosen professions and to stretch beyond local, state and national confines. Rather than embracing a technical rationality and following a one-size-fits-all approach, the AHEP flipped curriculum approach actively supports transformational engagement. Transformative learning is not new in health professional education. It is regarded as a pedagogical tool for the 21st century (48) therefore used in medicine and nursing because of its value in “producing enlightened change agents” (11). Accordingly, the UTAS AH programs use learning, teaching, and assessment strategies across the intended, enacted and experienced curriculum (27) to achieve core competencies for effective interprofessional collaboration and to challenge the dominant biomedical status quo that continues to prevail in healthcare. We aspire to create graduates who are adept in critical analysis for the creative adaptation of resources to address local health priorities.

The SHS continues to grapple with the problem of how the new AH programs can be organized to ensure all education assets and outcomes can be brought to bear on meeting the needs of Tasmania while balancing the need to ensure the program goals are achieved, quality maintained, and the affordability of program implementation sustained. There are already tangible benefits emerging from the AHEP, however, more work is required to continue to deliver on all program objectives, which can be achieved with ongoing concerted effort and collaboration between the University and various entities across the state who are partnering together to build statewide AH capacity.

## Conclusion

As the world becomes ever more interconnected, the interest in global public health grows and this signals a shift in the purpose of higher education. To improve public health and effectively prepare AH graduates for safe, quality, and agile practice across a range of contexts, programs of study are needed that are practical, raise a future health workforce, and graduate a generation of people that can transform systems, communities, and regions. Designing programs to prepare for the unpredictability of practice is challenging when curriculum must also respond to various education drivers. Curricula has become swollen with lectures and units of study with less time for independent thought, inquiry and study to prepare for professional practice. Innovation in education is now needed to think beyond what we are currently doing and find new ways to improve the quality and productivity of student learning. Design thinking as a curriculum design methodology is proving critical to SHS moving the AHEP forward as we continue to rethink how to prepare an AH health workforce to enable improved public health outcomes.

## Data availability statement

The original contributions presented in the study are included in the article/supplementary material, further inquiries can be directed to the corresponding author.

## Author contributions

Data collection and design of the Allied Health Expansion Program was led by NB and LD in collaboration with KS and PH. LD made substantial contributions to the conception or design of the original manuscript, including acquisition, analysis, and interpretation of data. AH and SJ provided assistance with framing, interpretation of findings, and subsequent revisions. All authors provided critical review and approved the final submission.
